# Clinical Lectures on Diseases of the Ear

**Published:** 1871-08

**Authors:** E. L. Holmes

**Affiliations:** Professor of Ophthalmology and Otology in Rush Medical College, Surgeon to the Chicago Charitable Eye and Ear Infirmary


ARTICLE IV.—*Clinical Lectures on Diseases of the Ear.* By E. L. HOLMES, M.D., Professor of Ophthalmology and Otology in Rush Medical College, Surgeon to the Chicago Charitable Eye and Ear Infirmary.

(Continued from page 439.)

PHYSIOLOGY OF THE EAR—SOUND.

*Gentlemen:*

You are familiar with the fact that undulations of the air set in vibratory motion the membrana tympani. The vibrations of this membrane produce, through the ossicles, microscopic waves in the fluid of the labyrinth, which beat against the fibres of the auditory nerve, producing the sensation we call sound.

You have almost a perfect, though rough, illustration of the mechanism of hearing, if you place the tip of a small syringe filled with water, in a small glass of water, in which float coarse particles of some dark powder. Slight but rapid motions of the piston will cause delicate currents in the water rendered visible by the motions of the particles floating in it.

To comprehend, as far as possible, the wonderful delicacy of the apparatus we have described, we must endeavor to conceive precisely what takes place in the ear, the ordinary medium of sonorous waves, when sound is produced.

A wave of sound, however originated, is a portion of the atmosphere in a state of condensation, followed by another portion in a state of rarefaction.

To picture in our minds these waves, which we cannot see, let us turn to a very familiar phenomenon.

A solid body dropped, for instance, into a placid sheet of water, causes waves to pass in circles from their origin to the shore. In this case a wave is that portion of water embraced in one elevation and one depression, its height and length depending on the size of the body producing it. This holds true in case of the mightiest billow of the ocean, or the tiniest ripple set in motion by the finest grain of sand.

You should bear in mind that atmospheric waves commence at a point and extend, not in circles as do the waves of fluids, but away from this point in spheres, consequently in whatever direction a person may be in reference to the origin of the sound, a portion of the sound wave must enter the ear.

When a series of waves in sufficiently rapid succession, whether caused by the vibration of a tuning fork, a stretched cord, or by any wind instrument, strikes against the membrana tympani, we have the sensation of a "tone;" the more numerous the vibrations, the higher the pitch of the tone.

The octave of any musical tone is produced when the number of vibrations causing the tone is doubled.

It is worthy of special remark that the human ear has no perception of a tone produced by vibrations exceeding about thirty-eight thousand in a second. Recur once more to the sheet of water I have already taken to illustrate, by its visible waves, the invisible waves in the air.

You have undoubtedly often observed that numerous small bodies dropped in regular and rapid succession into the water produced a series of small waves following each other in the same regular succession in which the bodies fell. You have also all observed on the surface of large waves smaller waves, and over these smaller waves ripples, all moving at the same instant. The eye is limited in its power of distinguishing the infinite gradations in the series of waves which absolutely exist and roll, the smaller over the larger, when a very large series of bodies, varying in size, from that, for instance, of a large cannon ball to that of a grain of sand, fall within a few seconds into water.

Now picture in your minds, if possible, what occurs in the air during the performance of a piece of music by a large orchestra. The various bass tones reach the ear in waves measured in length by several feet — and varying in length according to their pitch; in these long waves also move the short waves produced by the instruments of a high pitch. All these waves, so infinite in number and various in length, not only enter the ear at the same instant with unvarying regularity, but do so even though they cross each other in every direction, as when we stand in the centre of a circle, the orchestra being arranged at the circumference.

It will assist us in comprehending these phenomena and their relation to the ear, by turning for a moment to some of the phenomena of light. As sound is produced by undulations in the air (usually), so light, as now generally believed, is caused by undulations in (a hypothetical) ether.

As light may be reflected concentrated by convex lenses or by concave mirrors, so sound may be concentrated by concave surfaces or by suitable lenses. You have seen the phenomenon of vision illustrated by the photographer's camera, on the ground glass screen of which the images of objects before it are formed.

You have repeatedly seen the experiment, in which the camera has been filled with smoke, when not only the image on the screen was seen, but also the cones of light, their bases and apices, being clearly visible. You have also seen how these cones may be lengthened or shortened, by concave or convex lenses, their form changed by cylindrical (astigmatic) lenses, and their direction modified by prisms placed before the object glass of the camera.

In this manner we have visible before us, in miniature, suspended in the smoke, the cones of light radiating from every object. In fact, the camera not only presents an image of all objects before it, but also, on a reduced scale and reversed, every pencil of light emitted from these objects.

I have digressed in this manner to impress, if possible, upon your minds just what occurs in the ear. If it were possible to look into the labyrinth, and if the eye were sufficiently delicate, we should witness in the fluid of the labyrinth minute waves and ripples, which would be, as it were, reduced miniatures of every tremulous motion in the air, caused by the busy hum of human life, by the winds moving through the trees, by the fall of rain, the cry of animals, and notes of insects. All this infinitude of waves in the air finds each its individual counterpart accurately transferred to these few drops of fluid in the ear.

Thus far, in our studies of the anatomy and physiology of the ear, we have considered a few demonstrable facts. I will not lead you through the various theories which have been devised to explain the functions of different portions of the labyrinth and of the middle ear.

The theory of Helmholtz, however, regarding the cochlea is so beautiful, and supported by so many facts, that I cannot omit allusion to it here.

The portion of the auditory nerve distributed to the cochlea, is divided into a very large number of fine fibres, which may, not without propriety, be compared to the strings of a piano.

You are familiar with the fact that a musical tone, sounded either with the voice or some musical instrument over an open piano, causes the same tone to be repeated. Certain strings give this tone, while all the others are silent. Change the tone, the former strings will be silent, while others give the response.

In accordance with the theory of Helmholtz, low tones are distinguished by certain fibres alone, and high tones by other fibres of the cochlea nerve.

It is a curious pathological fact that there are patients to whom the lowest tones of the scale are absolutely inaudible, while all ordinary sounds and tones are accurately heard; other patients are totally deaf to the highest tones of the scale, because, according to this theory, the nervous fibres which distinguish the base or high tones are paralyzed.

From what has thus far been shown, you can appreciate to what extent sound is produced by mechanical means, and to what extent the auditory apparatus is strictly mechanical.

From this you will not be surprised to learn that deafness, in a very large proportion of cases, is produced by impaired motion of the stapes. Chronic inflammation of the middle ear causes thickening of the membrane which surrounds the minute bones, which forms the "packing" of the stapes, which constitutes the membrane of the foramen rotundum, and of less importance, perhaps, the membrana tympani.

In fact, gentlemen, I do not think it is in any way unscientific to compare the ear, in a very great majority of cases of chronic inflammation, to a syringe in which the motions are obstructed or arrested by tightness around the handle, or by too close packing around the piston.

In our future lectures we shall confine our investigations to the answer of practical questions regarding the causes, diagnosis and treatment of the more common diseases of the ear.

